# Supplementary material for: Deciphering genetic susceptibility to clear cell renal cell carcinoma
Source: Commun Biol. 2025 Dec 1;9:32. doi: 10.1038/s42003-025-09297-w (PMC12780051; doi:10.1038/s42003-025-09297-w)
Supplement: Supplementary file 2 — Supplementary Figs. [file 42003_2025_9297_MOESM2_ESM.pdf]

A

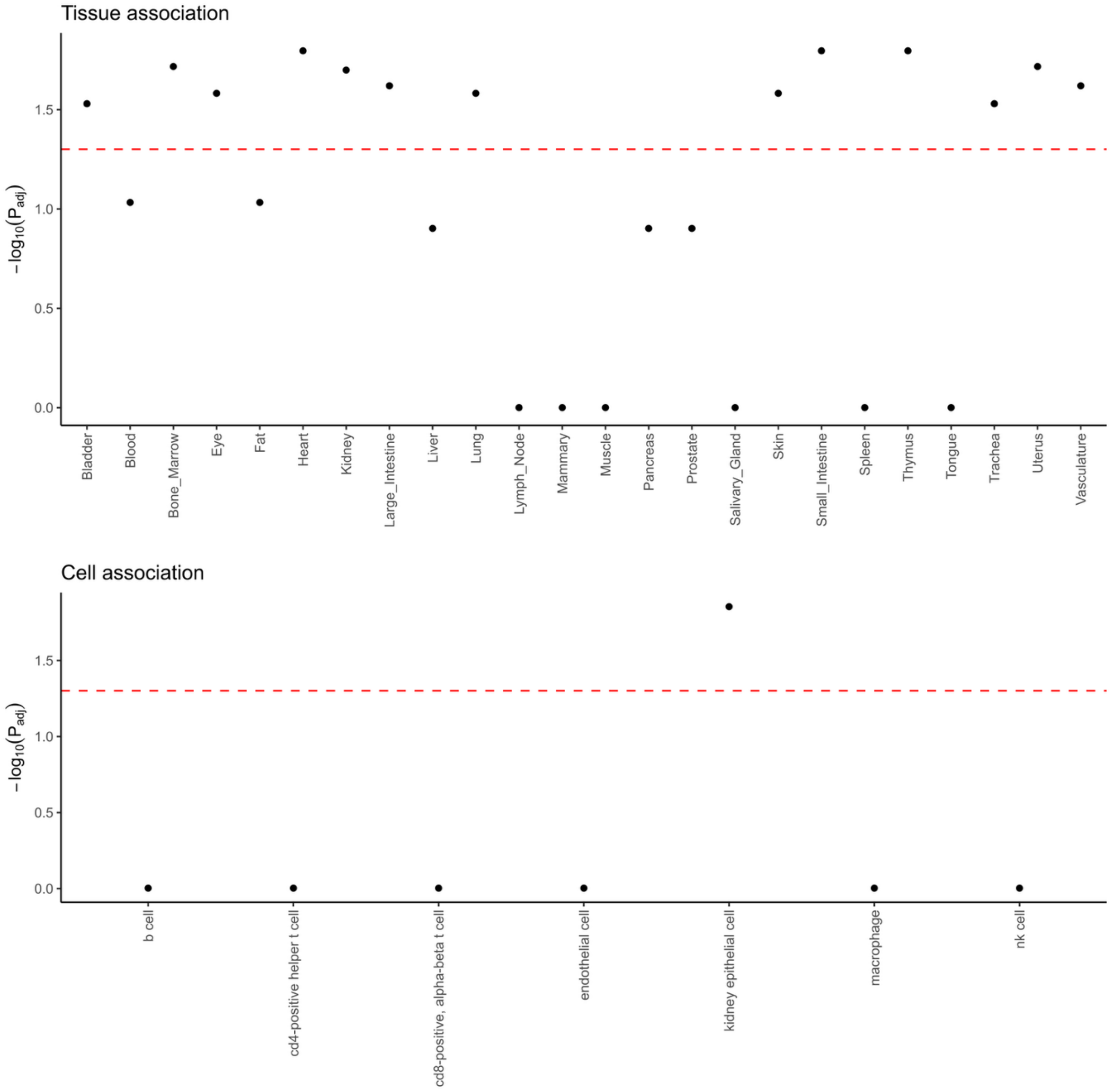
Tissue association
$-\log_{10}(P_{adj})$
0.0
0.5
1.0
1.5
Bladder
Blood
Bone_Marrow
Eye
Fat
Heart
Kidney
Large_Intestine
Liver
Lung
Lymph_Node
Mammary
Muscle
Pancreas
Prostate
Salivary_Gland
Skin
Small_Intestine
Spleen
Thymus
Tongue
Trachea
Uterus
Vasculature
Cell association
$-\log_{10}(P_{adj})$
0.0
0.5
1.0
1.5
b cell
cd4-positive helper t cell
cd8-positive, alpha-beta t cell
endothelial cell
kidney epithelial cell
macrophage
nk cell

B

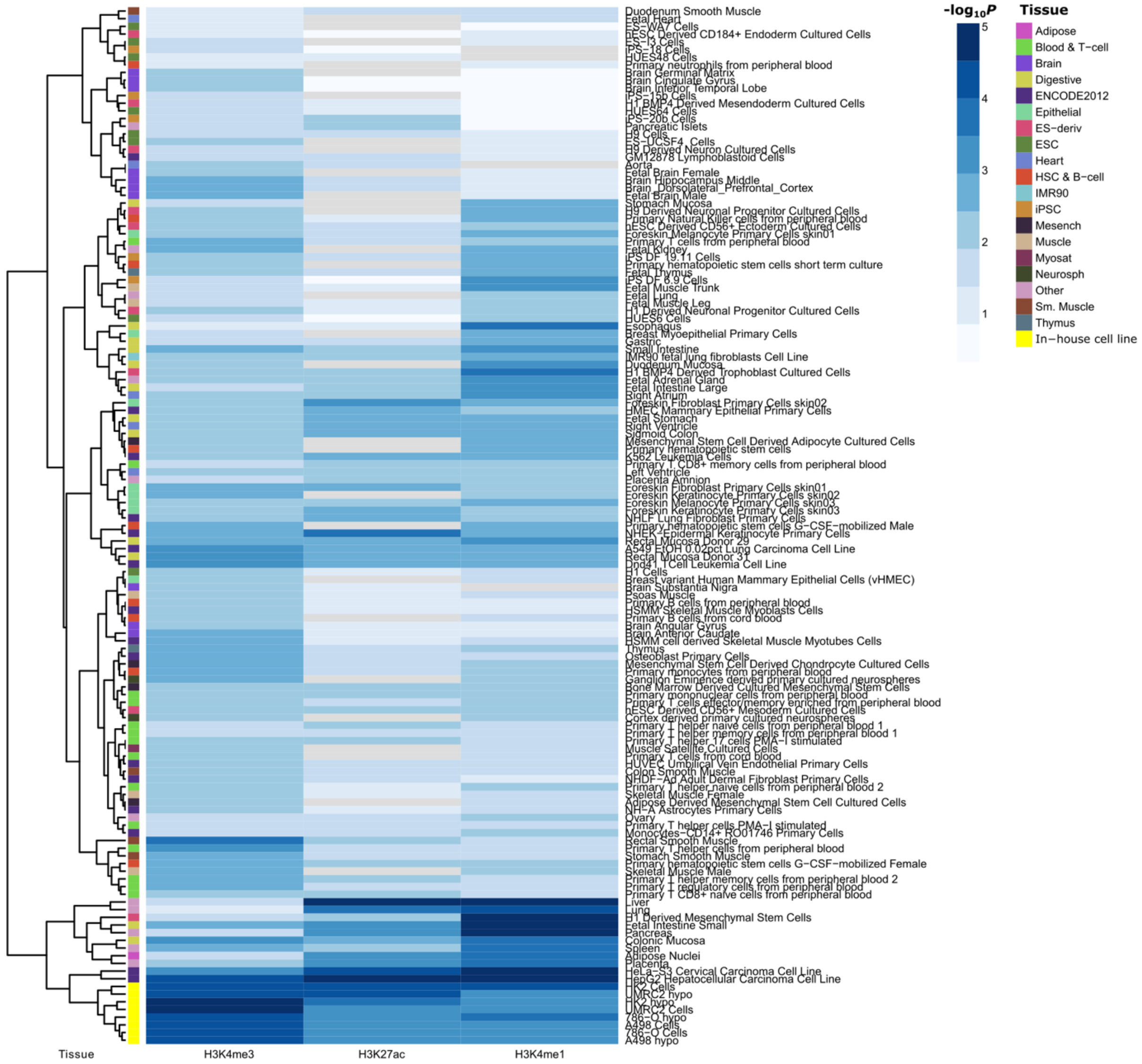

Supplementary Figure 1: (A) Enrichment of the GWAS using scDRS in the Tabula Sapiens dataset, investigating tissues (top) and individual cell types (bottom) showing enrichment in kidney epithelium. Plotted *P*-values were Benjamini-Hochberg corrected. The red line indicates a *P*-value of 0.05 (B) Enrichment of H3K4me1, H3K4me3, and H3K27ac in the NIH Roadmap Epigenome Project data in a series of cell types, as well as RCC cell lines. Colour is scaled to the -$\log_{10}$(*P*-value) of the enrichment.

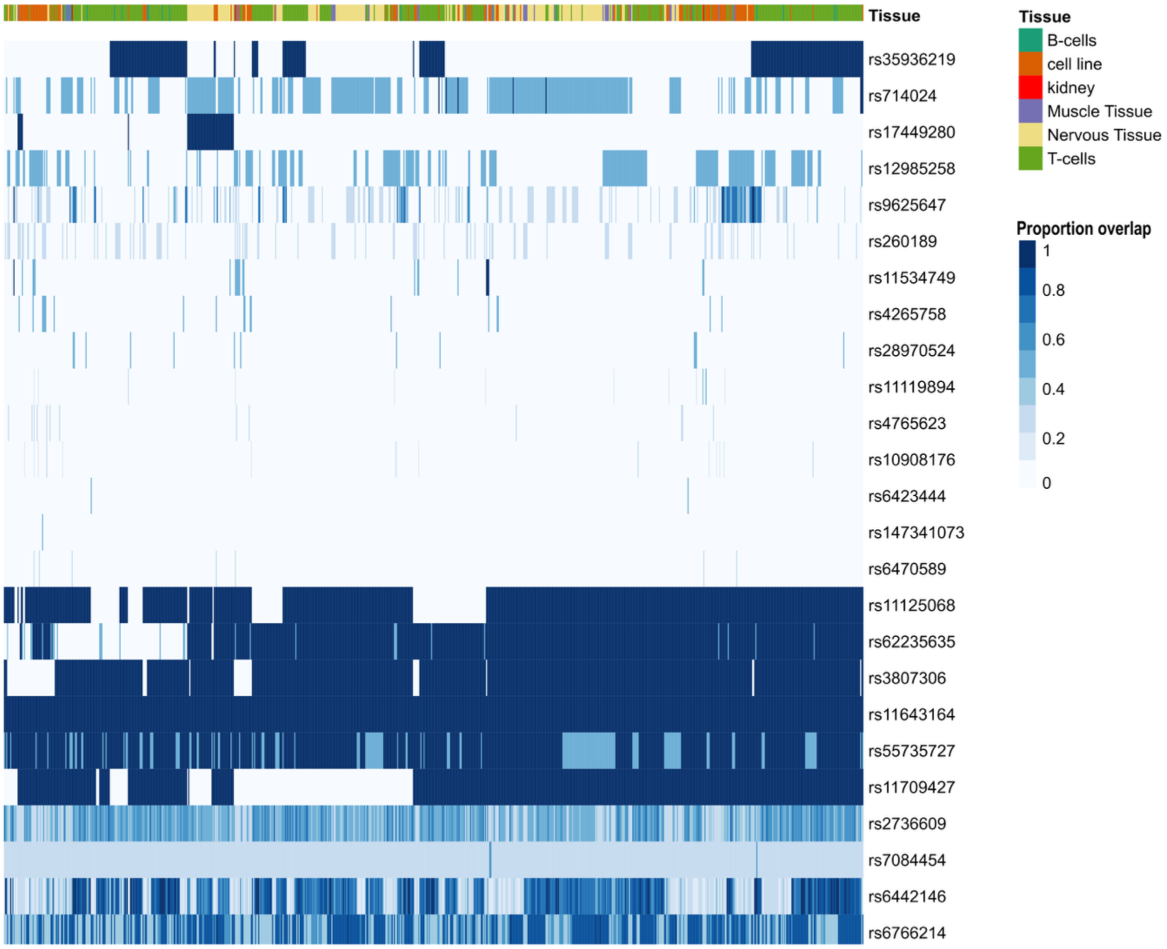

Supplementary Figure 2: Heatmap illustrating common ABC enhancer-gene predictions across 811 cell lines and adult tissues in ENCODE, as compared to the ccRCC cell line 786-O. The top bar indicates the tissue type for each sample. Each row represents a ccRCC risk variant with an enhancer-gene association, and the colour intensity in the heatmap shows the proportion of overlap where the same enhancer with the same target gene were predicted in the other tissues.

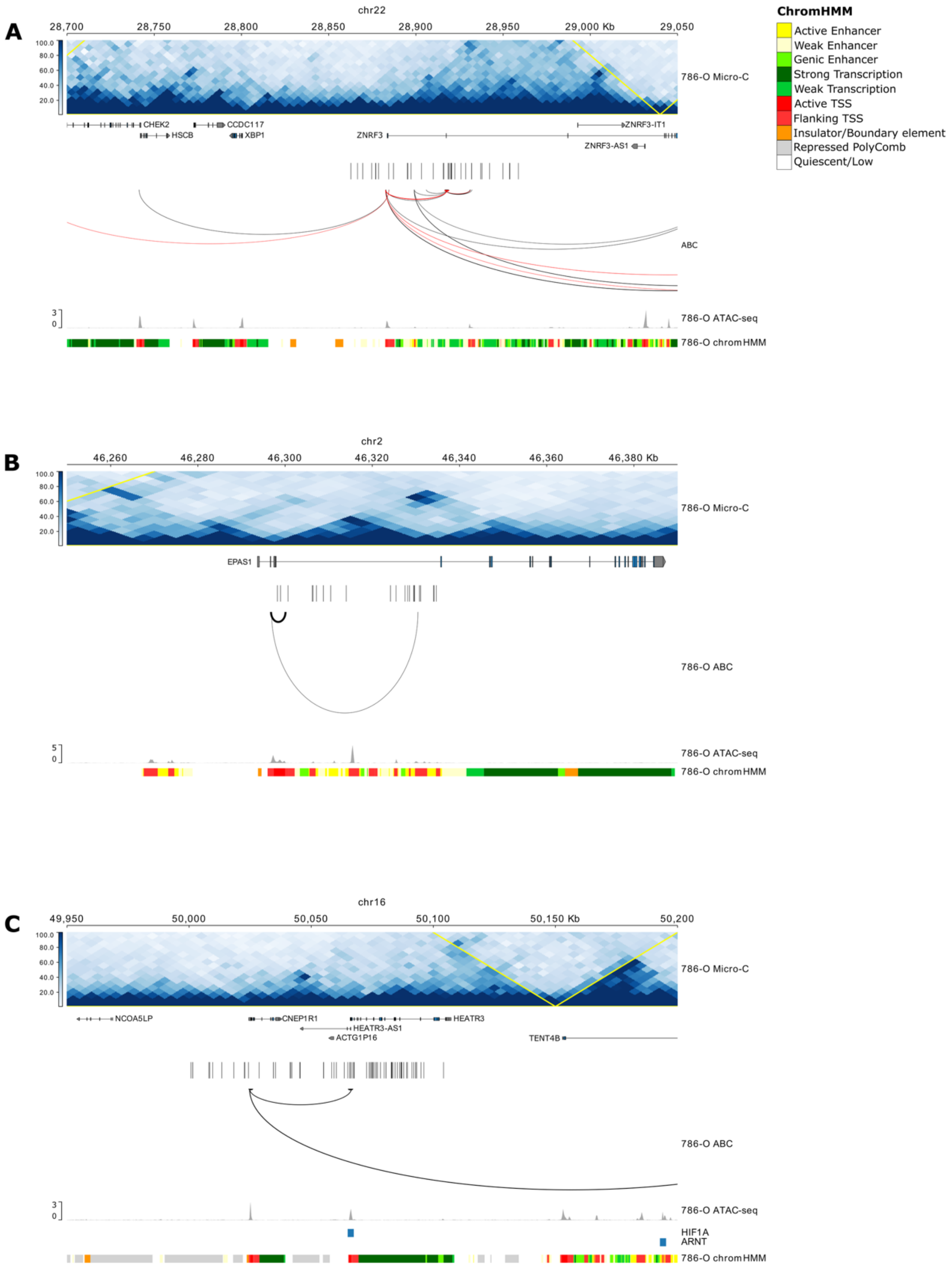

Supplementary Figure 3: Region plots showing the detailed annotation of (A) 22q12 (*CHEK2*), (B) 2p21 (*EPAS1*), and (C) 16q12 (*HEATR3*). For each sub-figure from the top are the micro-C contact map with predicted topological associated domains (TAD) overlaid in yellow, gene models, GWAS variants ($r^2 > 0.8$ to lead variant), micro-C chromatin loop, ATAC-seq peaks, and the predicted chromHMM states. In addition, for sub-figure A, the loops from HK-2 are shown in red. For clarity, only protein-coding genes are shown. Coordinates are in GRCh38.

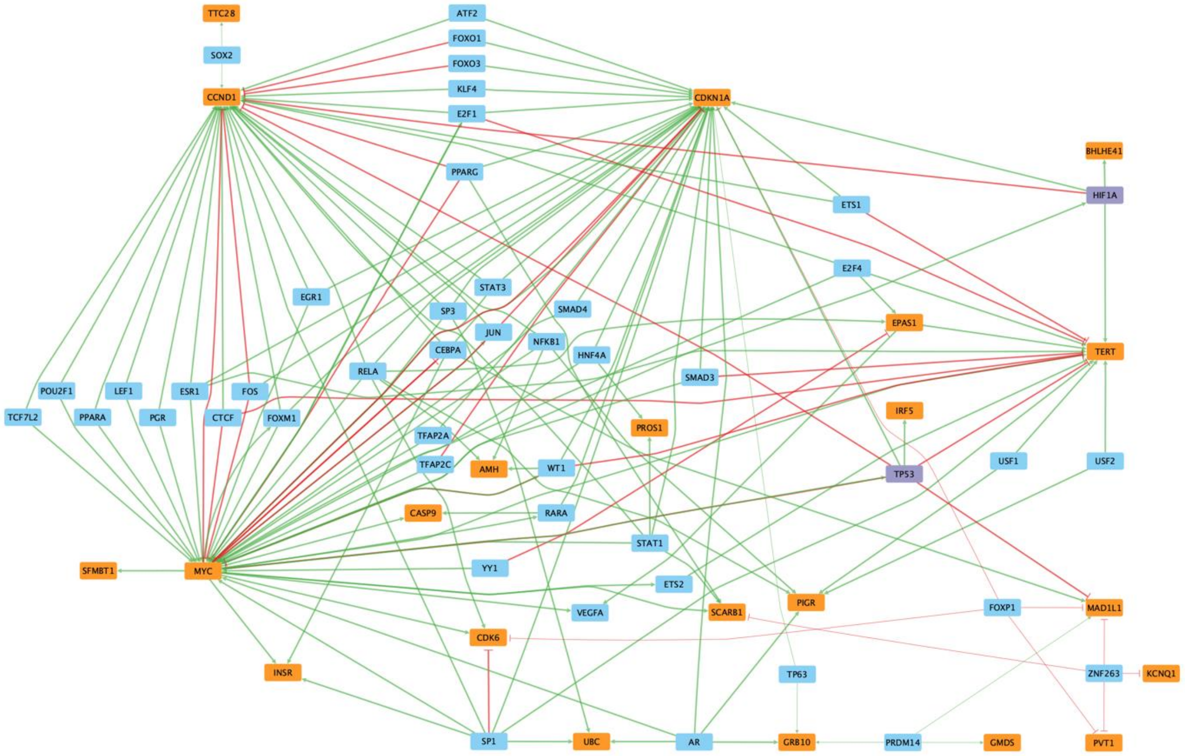

Supplementary Figure 4: Regulatory network of the identified putative target genes. Green lines indicate stimulation and red lines indicate repression. Line thickness is indicative of the confidence of the interaction. Genes present in the gene list are coloured orange, and genes in purple are those identified from somatic studies. Regulatory interaction data were obtained from the OmniPath/DoRothEA based on associations derived from cancer cells.
