# Supplementary material for: Deciphering genetic susceptibility to clear cell renal cell carcinoma
Source: Commun Biol. 2025 Dec 1;9:32. doi: 10.1038/s42003-025-09297-w (PMC12780051; doi:10.1038/s42003-025-09297-w)
Supplement: Supplementary file 3 — Description of Additional Supplementary Files [file 42003_2025_9297_MOESM3_ESM.pdf]

Description of Additional Supplementary Files:

File: Supplementary Data 1

Description: Table of ccRCC risk variants and annotations

File: Supplementary Data 2

Description: Results from the statistical finemapping results

File: Supplementary Data 3

Description: Summary of MPRA results from Kellman et al (2025)

File: Supplementary Data 4

Description: Top ABC results for each locus, across multiple cell lines

File: Supplementary Data 5

Description: Differential expression results from RNA-seq of putative target genes

File: Supplementary Data 6

Description: List of enriched pathways

File: Supplementary Data 7

Description: Mendelian Randomisation results of ccRCC and LTL

File: Supplementary Data 8

Description: Colocolisation results of ccRCC and LTL

File: Supplementary Data 9

Description: Results from polygenic risk score association with clinical traits

File: Supplementary Data 10

Description: OncoScore results on putative target genes

File: Supplementary Data 11

Description: Literature triplets on putative target genes from MELODI Presto

File: Supplementary Data 12

Description: Analysis of gene draggability on putative genes

File: Supplementary Data 13

Description: GWAS results from other ancestries

File: Supplementary Data 14

Description: Data used to generate Figure 3, and Supplementary Figures 1A, 1B, and 2
